# The Genetics of Keratoconus

**DOI:** 10.4103/0974-9233.75876

**Published:** 2011

**Authors:** Dorota M. Nowak, Marzena Gajecka

**Affiliations:** Institute of Human Genetics, Polish Academy of Sciences, Poznan, Poland, Strzeszynska 32, 60-479 Poznan

**Keywords:** Genetics, Keratoconus, Keratoconus Loci

## Abstract

Keratoconus (KTCN) is non-inflammatory thinning and anterior protrusion of the cornea that results in steepening and distortion of the cornea, altered refractive error, and decreased vision. Keratoconus is a complex condition of multifactorial etiology. Both genetic and environmental factors are associated with KTCN. Evidence of genetic etiology includes familial inheritance, discordance between dizygotic twins, and association with other known genetic disorders. Several loci responsible for a familial form of KTCN have been mapped; however, no mutations in any genes have been identified for any of these loci. This article focuses on the genetic aspects. In addition, bioinformatics methods applied in KTCN gene identification process are discussed.

## INTRODUCTION

Keratoconus (KTCN) is noninflammatory thinning and anterior protrusion of the cornea that results in steepening and distortion of the cornea, altered refractive powers, and altered visual acuity. In more advanced cases, corneal scarring from corneal edema and decompensation further reduces visual acuity. Symptoms are highly variable and depend on the stage of progression of the disorder.^1^,^2^ There is a large variance in the reported prevalence of KTCN ranging from 8.8 to 54.4 per 100,000.^3^–^5^ This variation is in part due to the different diagnostic criteria used in various studies.^3^–^5^ KTCN is known to affect all ethnicities. For example, incidence of KTCN in Asians is 25 per 100,000 (1 in 4000) per year, compared with 3.3 per 100,000 (1 in 30,000) per year in Caucasians (*P* < 0.001).^6^ This article focuses on the genetic aspects of this disease and does not cover clinical management.

## KERATOCONUS AS A MULTIFACTORIAL DISEASE

Genetic disorders can be divided into Mendelian and multifactorial diseases. In Mendelian diseases, a mutation in a single gene can cause a disease that is inherited according to Mendel’s laws. Mendelian disorders are rare and occur in a population with frequencies lower than 0.05%.^7^ In contrast, in complex or multifactorial diseases, changes in numerous genes and environmental factors are responsible for the disease development and progression.

KTCN is a complex condition with multifactorial etiology. Both genetic and environmental factors are associated with KTCN. Evidence of genetic etiology includes the familial inheritance, discordance between dizygotic twins, and its association with other known genetic disorders. Environmental factors include contact lens wear, chronic eye rubbing, and atopy of the eye.^1^,^8^,^9^

Although numerous studies of KTCN are reported, identifying the genetic factors remains elusive. Phenomena, such as gene–gene interactions, genetic heterogeneity, reduced penetrance, or phenocopy, that are frequently observed in complex diseases significantly influence genetic factors and the identification process. Genetic heterogeneity consists of allelic heterogeneity (different mutations in the same locus) and/or locus heterogeneity with different loci producing the same phenotype. To date, locus heterogeneity has been extensively observed in KTCN studies. However, sequence variants in *VSX1* could be discussed as an example of allelic heterogeneity.^10^ Reduced penetrance means that some individuals with a predisposing genotype may not develop disease; this could be due to environmental or random interactions. In KTCN, penetrance is estimated to be 0.5–0.8 in linkage analysis.^11^–^13^ A phenocopy is an individual who has disease phenotype due to environmental factors, although he/she does not have a predisposing genotype. In KTCN linkage studies, phenocopies are estimated to be 0.001-0.01.^12^,^13^

Linkage analysis and association studies are the two main approaches used to identify the causative genes. Linkage analysis identifies chromosomal region(s) associated with the disease and the gene(s) mapped to that region(s). Linkage analysis has been extensively used in the KTCN gene mapping in different populations [Table 1].^11^–^17^ One method to identify the causative gene(s) in a population under study is to focus on the number of families with multiple affected individuals, preferably in two or more generations, which form a homogenous study group, derived from the same population, without racial differences and other factors that may affect heritability. The minimum subject requirement is a single pair of affected siblings, however, both affected and unaffected individuals are involved in the analysis. Affected individuals would inherit the affected portion of a chromosome from their parent(s). Chromosomes or pieces of chromosomes that were inherited equally by affected and unaffected family members (ie, segregating randomly according to the Mendelian inheritance) are not associated with disease. Linkage analysis, as a model-based method, requires a frequency of disease allele, penetration, and phenocopy, which in the case of KTCN are usually unknown. A solution could be a model-free method in which pairs of affected relatives are analyzed. In this type of analysis identity-by-descent (IBD) distribution is used, and this determines probabilities of alleles inherited from a common ancestor by pairs of relatives. For affected sib-pairs, the expected probabilities for sharing 0, 1, and 2 alleles, IBD are 0.25, 0.5, and 0.25, respectively.

**Table 1:** Identified loci for keratoconus genes

Locus	Methods	Associated disorders	Author
1p36.23-36.21	Linkage	-	Burdon KP, 2008
2p24	Linkage	-	Hutchings H, 2005
2q13	Association	-	Kim S, 2008
3p14-q13	Linkage	-	Brancati F, 2004
5q14.3-q21.1	Linkage	-	Tang YG, 2005
5q21.2	Linkage	-	Bisceglia L, 2009
5q32-q33	Linkage	-	Bisceglia L, 2009
7q21.11	Association	-	Burdon KP, 2010
7q36.1	Association	-	Burdon KP, 2010
8q13.1-q21.11	Linkage	-	Burdon KP, 2008
9q32	Association	-	Burdon KP, 2010
9q34	Linkage	-	Li X, 2006
13q32	Linkage	-	Gajecka M, 2009
14q11.2	Linkage	-	Bisceglia L, 2009
14q24.3	Linkage	-	Liskova P, 2010
15q15.1	Linkage	-	Bisceglia L, 2009
15q22.33-24.2	Linkage	Anterior polar cataract	Hughes AE, 2003
16q21	Association	-	Burdon KP, 2010
16q22.3-q23.1	Linkage	-	Tyynismaa H, 2002
17p13	Linkage	Leber congenital amaurosis (LCA4)	Hameed A, 2000
20q12	Linkage	-	Fullerton J, 2002

Genetic heterogeneity causes difficulties in linkage analysis. In allelic heterogeneity, despite different polymorphisms in a gene, all families will show linkage to the same chromosomal region.^18^ On the contrary, locus heterogeneity may cause serious problems in gene(s) identification; in particular, in families from the same population, different genetic loci may be involved. Usually large families are needed to obtain robust linkage evidence.^18^ Still, traditional linkage studies concentrate on each locus separately. However, in complex disease, where more than one gene is considered, gene–gene interaction should also investigated. One of the attempts to present the disease more realistically in a linkage analysis is a method allowing for analyzing two distinct loci simultaneously. Such analysis performed in an Australian pedigree by Burdon *et al*., identified 1p36.23–36.21 and 8q13.1–q21.11 loci.^19^ First, the analysis revealed two separate loci with LOD scores of 1.94 and 1.96. However, analyzing two distinct loci concurrently increased the LOD score to 3.4.

Despite many linkage studies on complex diseases, loci identified as being related to the disease are rarely confirmed by further studies. To date, only one KTCN locus, 5q21.2, previously reported by Tang *et al*.^17^ has been replicated by Bisceglia *et al*.^14^

In association analysis, a relationship between allele or genotype of genetic marker and a trait is examined. Direct or indirect genetic associations are performed. In a direct association, analysis of polymorphism as the causative factor of the disease is conducted. In contrast, the indirect association analysis does not require that the analyzed marker is responsible for the disease; it must merely be in linkage disequilibrium with the causative polymorphism. Association analysis for KTCN is less frequently applied than linkage study. Association study to assess sequence variants in selected candidate genes is one of the possible applications. Another is genome-wide scan, genome-wide association study (GWAS). GWAS allows the analysis of the whole genome in one experiment. A few candidate KTCN genes were identified in association study, including *IL1B*,^20^ *CDH11, NUB1, COL27A1*, and *HGF*.^15^

There are many limitations of association or linkage studies of complex diseases. One limitation of linkage studies is the size of the genetic effect. Sample sets should be two or three orders of magnitude larger for small or moderate effects to provide enough power of analysis.^18^,^21^,^22^ Genome-wide association methods with case–control cohort allow collection of larger patient group than family-based studies, which increases the power of analysis. This method makes it possible to identify genes with lower effect, but it partially explains heritability. For example, heritability of human height is established as high as 80%, but 40 identified loci explain only 5% of heritability.^23^ Most probably there are numerous unidentified variants influencing human height. On the other hand, very rare variants with high effect may be responsible for phenotype production. However, based on the hypothesis “common disease, common variant,” most of today’s commercial microarrays use only single nucleotide polymorphisms (SNPs) observed in 1%–5% of the general population. In contrast to case–control and case only analyses, the family is the best model to identify a rare sequence variant. However, it should be expected that the locus identified in one family only, will not be replicated in the general population. For example, the analysis of families from the United Kingdom with high myopia showed that locus MYP3 could be responsible for myopia in only 25% of the analyzed families.^24^ Even in a homogeneous population it is difficult to find strong linkage evidence. For example, in the linkage analysis in Ecuadorian families with KTCN, only one large family presented with significant LOD at locus 13q32.^12^

## FAMILIAL KERATOCONUS

Although the majority of patients presenting to ophthalmologists with KTCN have a sporadic form of the disease, there is growing evidence of familial KTCN and the involvement of genetic factors. Previous prospective studies revealed that relatives of patients with KTCN had a high prevalence of undiagnosed KTCN.^25^,^26^ If complete slit-lamp examination, refraction, and corneal topography were performed, 11%–14% of apparently unaffected relatives of patients with KTCN were diagnosed with KTCN changing the KTCN classification from sporadic to familial.^25^,^26^

Ninety percent of pedigrees with familial KTCN display an autosomal dominant inheritance with reduced penetrance.^27^,^28^ Other modes of inheritance have been described, including autosomal recessive mode in families with children of consanguineous parents.^29^,^30^

Numerous loci have been mapped in KTCN families and research is ongoing to identify causative genes involved in KTCN development and progression.^11^,^13^,^16^,^17^,^27^,^31^,^32^ A locus for autosomal dominant KTCN was mapped in Finnish families to 16q22.3–q23.1.^31^ Another locus on 15q22.33–24.2 was reported in a three-generation Northern Irish family in which the affected individuals presented with combined early-onset autosomal dominant anterior polar cataract and KTCN, and candidate genes *CTSH, CRABP1, IREB2*, and *RAS-GRF1* were excluded.^27^ Other combined disease phenotypes were mapped to 17p13 in a two-generation Pakistani family with autosomal recessive Leber congenital amaurosis and KTCN.^33^ In a two-generation Italian family with autosomal dominant KTCN, a locus on chromosome 3 was identified at 3p14–q13.^11^ However, mutation analysis of *COL8A1*, the candidate gene located within the genetic region, did not show any pathogenic mutation.^11^ An additional locus for KTCN was identified on 5q14.3–q21.1 in a four-generation autosomal dominant Caucasian pedigree.^17^ A locus on 2p24 was identified in a heterogeneous population of 28 families recruited in France, Spain, and Guadeloupe, of European, Arab, and Caribbean-African descent.^13^ Recently genome-wide linkage analysis performed in autosomal dominant multigenerational KTCN families from Ecuador provided evidence for a novel locus on 13q32.^12^ Also, evidence of linkage for KTCN to chromosomes 4, 5, 9, 12, and 14 has been obtained from a genome-wide linkage analysis with data from KTCN sib-pair families of white or Hispanic origin.^34^ Moreover, Bisceglia *et al*. found several regions suggestive of linkage, at the chromosomal regions 5q32–q33, 5q21.2, 14q11.2, and 15q2.32, and a locus replication at 5q21.2.^14^ A genome-wide linkage scan in a large Australian pedigree was conducted and two regions of linkage at 1p36.23–36.21 and 8q13.1–q21.11 were identified.^19^ Recently, a GWAS was performed and with several SNPs in a region on chromosome 11 linked to KTCN.^15^ In addition, evidence for KTCN susceptibility locus on chromosome 14q24.3 has been reported.^35^ However, to date, no mutations in any genes have been identified on any of these KTCN loci.

The family ascertainment, including both affected and unaffected individuals, is a complicated and time-consuming process in which each study participant undergoes a complete ophthalmic evaluation. In KTCN, subclinical forms of the disease may exist, hence, accurate inclusion criteria should be established prior to the linkage analysis to minimize misclassification. Currently, computerized videokeratography allows the detection of early forms of KTCN, to some extent solving the problem of phenotype misclassification. However, some families display a specific inheritance pattern with reduced penetrance, which may be a confounding factor in data interpetation.^12^

## KERATOCONUS GENE

To date, no mutations in any genes have been identified for any of the discussed KTCN loci. Other reports indicate mutations in SOD1 (MIM 147450, locus 21q22.11) and *VSX1* (KTCN1, MIM605020, locus 20p11.2) genes as enrolled in the etiology of KTCN, however, none of the subsequent studies have confirmed role of these changes.^12^,^36^,^37^ Other candidate genes for KTCN, such as *COL6A1, COL8A1, MMP9*, and *MMP2*, have been examined and excluded as causative genes.^16^,^37^,^38^ These results support the theory of multiple gene involvement rather than a single major gene in the development and progression of KTCN.

## MONOZYGOTIC TWIN STUDIES

Monozygotic twin studies constitute an adequate research model to evaluate genetic and environmental factors in the disease pathogenesis. The higher the rate of concordance between the monozygotic twins, the greater the evidence for primary genetic causation rather than environmental etiology. Also, if concordance is greater between monozygotic compared with dizygotic twins, genetic factors likely play a key role in the disease phenotype.

In general, KTCN studies have reported monozygotic twins concordant, rather than discordant for KTCN, especially when twins were examined with modern computerized videokeratoscopy.^39^–^41^ This concordance supports the evidence of heredity as a genetic factor in the etiology of KTCN.^41^ Variation in corneal topography between a pair of monozygotic twins can be explained by individual genetic susceptibility and the influence of environmental factors. In cases discordant for KTCN reading, eye rubbing and hormonal influences were suggested as environmental factors involved in the etiology of KTCN.^42^ Furthermore, phenotypic differences in monozygotic twin pairs may reflect genotypic differences resulting from somatic changes occurring during development.^40^ Consequently, cases discordant for KTCN could be phenotypically monozygotic twins not identical genetically in all tissues, therefore, genotypically discordant.^40^

## ASSOCIATION OF KERATOCONUS WITH OTHER KNOWN GENETIC DISORDERS

More than two dozen syndromes are associated with KTCN, including Down syndrome,^43^ Leber congenital amaurosis,^44^ connective tissue disorders, including osteogenesis imperfecta,^45^ GAPO syndrome,^46^ and some subtypes of Ehlers–Danlos syndrome.^47^,^48^

Down syndrome has a strong association with KTCN, with a reported prevalence ranging from 0.5% to 15% (10- to 300-fold that of the normal population).^1^,^43^,^49^–^51^ Additionally, an association between KTCN and autosomal recessive Leber congenital amaurosis is observed in up to 30% of patients.^44^ Recently, an association of mutations in *CRB1* gene with KTCN in patients with Leber congenital amaurosis has been reported.^52^ There are several reports linking KTCN and connective tissue diseases, such as subtypes of Ehlers–Danlos syndrome, osteogenesis imperfecta, mitral valve prolapse, and joint hypermobility, suggesting that KTCN is a localized manifestation of a mild connective tissue disorder.^48^,^53^,^54^

The association of KTCN with mental retardation is common. One study has shown that among 212 institutionalized mentally retarded individuals, there were 16 patients with KTCN (7.5%), eight of whom had unilateral disease.^55^ In a study by Kirby *et al*., siblings presented with severe mental retardation and bilateral KTCN.^29^ Another study from the Central Institution for the Mentally Retarded, in Klaebu, Norway, reported that nine of 30 patients had KTCN.^50^

## SUMMARY

The complexity of KTCN makes it difficult to identify factors influencing its development. Contact lens wear, chronic eye rubbing, and atopy of the eye, together with genetic etiology demonstrated by familial inheritance, discordance between dizygotic twins, and its association with other known genetic disorders have been extensively discussed by multiple research groups. However, to date, no diagnostic test was developed to predict or confirm KTCN in isolated or familial cases. Identification of genetic factors might allow to develop both specific diagnostic tests and KTCN gene therapy in the future. Furthermore, knowing the genetic basis of the disease, it will be possible to undertake research into medicines that allow some patients to avoid the need for a transplant.
